# Educational Technologies in Problem-Based Learning in Health Sciences Education: A Systematic Review

**DOI:** 10.2196/jmir.3240

**Published:** 2014-12-10

**Authors:** Jun Jin, Susan M Bridges

**Affiliations:** ^1^Faculty of EducationThe University of Hong KongHong KongChina; ^2^Centre for the Enhancement of Teaching and Learning/Faculty of EducationThe University of Hong KongHong KongChina

**Keywords:** systematic review, educational technologies, problem-based learning, medical education, health sciences, software, digital learning object, interactive whiteboard, learning management system

## Abstract

**Background:**

As a modern pedagogical philosophy, problem-based learning (PBL) is increasingly being recognized as a major research area in student learning and pedagogical innovation in health sciences education. A new area of research interest has been the role of emerging educational technologies in PBL. Although this field is growing, no systematic reviews of studies of the usage and effects of educational technologies in PBL in health sciences education have been conducted to date.

**Objective:**

The aim of this paper is to review new and emerging educational technologies in problem-based curricula, with a specific focus on 3 cognate clinical disciplines: medicine, dentistry, and speech and hearing sciences. Analysis of the studies reviewed focused on the effects of educational technologies in PBL contexts while addressing the particular issue of scaffolding of student learning.

**Methods:**

A comprehensive computerized database search of full-text articles published in English from 1996 to 2014 was carried out using 3 databases: ProQuest, Scopus, and EBSCOhost. Eligibility criteria for selection of studies for review were also determined in light of the population, intervention, comparison, and outcomes (PICO) guidelines. The population was limited to postsecondary education, specifically in dentistry, medicine, and speech and hearing sciences, in which PBL was the key educational pedagogy and curriculum design. Three types of educational technologies were identified as interventions used to support student inquiry: learning software and digital learning objects; interactive whiteboards (IWBs) and plasma screens; and learning management systems (LMSs).

**Results:**

Of 470 studies, 28 were selected for analysis. Most studies examined the effects of learning software and digital learning objects (n=20) with integration of IWB (n=5) and LMS (n=3) for PBL receiving relatively less attention. The educational technologies examined in these studies were seen as potentially fit for problem-based health sciences education. Positive outcomes for student learning included providing rich, authentic problems and/or case contexts for learning; supporting student development of medical expertise through the accessing and structuring of expert knowledge and skills; making disciplinary thinking and strategies explicit; providing a platform to elicit articulation, collaboration, and reflection; and reducing perceived cognitive load. Limitations included cumbersome scenarios, infrastructure requirements, and the need for staff and student support in light of the technological demands of new affordances.

**Conclusions:**

This literature review demonstrates the generally positive effect of educational technologies in PBL. Further research into the various applications of educational technology in PBL curricula is needed to fully realize its potential to enhance problem-based approaches in health sciences education.

## Introduction

As a modern pedagogical philosophy, problem-based learning (PBL) is increasingly being recognized as a major research area in student learning and pedagogical innovation in health sciences education. In contrast to traditional lecture-dominant teaching and learning approaches, inquiry-based approaches such as PBL prompt students to actively engage in knowledge construction and develop competencies across multiple contexts [1,2]. This review focuses on PBL instead of other distinct inquiry-based pedagogical approaches, such as discovery learning, experiential learning, and project-based learning. Given the high level of technological engagement of 21st century learners, a new area of research interest is examining the role of emerging educational technologies in PBL [3-5]. Therefore, the aim of this paper is to review new and emerging educational technologies in problem-based curricula with a specific focus on 3 cognate disciplines: medicine, dentistry, and speech and hearing sciences. The selection of these 3 related health sciences curricula is based on their level of current activity in the development and research of PBL [6-8]. Of particular interest to this review are studies investigating the role of such technologies in achieving PBL-related student learning outcomes of flexible knowledge, effective problem-solving skills, self-directed learning skills, collaborative teamwork skills, and intrinsic motivation [9,10].

Included studies are ones in which educational technologies have been adopted to support problem-based approaches to learning in both undergraduate and postgraduate programs. The types of technological innovations identified encompass such affordances as learning management system (LMS), specialist learning software (eg, CMapTools), immersive virtual environments (eg, SecondLife), and resources such as 3-dimensional (3D) anatomy models. Also of interest was the use of new hardware, such as interactive whiteboards (IWBs), and how these are combined to reshape new forms of learning in both synchronous, face-to-face “PBL 2.0” [3,11]. Additional studies are exploring the potential to initiate asynchronous models of PBL drawing on distance education needs and modes of delivery [12]. Such innovations draw on the potential of new technologies to provide a rich learning context with access to well-structured information and new spaces for knowledge collaboration [13]. However, although the field is growing and a few reviews have focused on e-learning innovation in health sciences and education [14,15], to date there is no existing systematic review of empirical studies on the usage of educational technologies in PBL in health sciences education.

We have identified 8 roles for technology in learning in the educational literature [16] relevant to identifying studies for inclusion in this review:

1. Access to and structuring of information
2. Curriculum platform
3. Communications media
4. Thinking tools
5. Rich contexts for learning
6. Collaboration spaces
7. A perspective toolkit
8. Scaffolding

The latter issue of scaffolding refers to situations in which experts offer assistance to learners in carrying out new tasks that learners would not be able to complete without support [17]. This issue has been debated in recent PBL and inquiry learning scholarship [18,19] with detractors indicating concerns that PBL does not provide sufficient scaffolding and that the open nature of the problems may add to cognitive load [18]. Proponents argue that PBL is highly scaffolded through strategies such as making disciplinary thinking and strategies explicit, embedding expert guidance, and structuring complex tasks thereby reducing cognitive load [19]. Open to further debate is whether the inclusion of technological affordances such as iPads, laptops, and simulations or variations of synchronous and asynchronous technology-rich delivery of PBL will support or detract from the scaffolding of learning.

Analysis of the studies reviewed will, therefore, focus on the effects of educational technologies in the PBL cycle while addressing the issue of scaffolding of student learning in particular both in face-to-face tutorials and during self-directed learning. The overarching goal is to provide new insights on how learners synthesize information from the multiple technologies employed in PBL at a time of pronounced educational innovation [13,20].

## Methods

### Focus Questions

Inspired by Cook and West’s approach [20] to conducting systematic reviews in medical education and existing review papers [21-24], the screening and classification process conducted is presented subsequently.

The focused questions addressed the population, intervention, comparison, and outcomes (PICO) model as recommended by Cook and West [20]. In addressing the issues above, the research questions addressed in this review are:

1. What effects of educational technologies on students and tutors have been identified in PBL-based applications?
2. How can educational technologies support digitally enhanced and interactive PBL in health sciences education?

### Eligibility Criteria

Eligibility criteria for the selection of studies for review were also determined in light of the PICO guidelines. The population was limited to postsecondary education, specifically in dentistry, medicine, and speech and hearing sciences, in which PBL was the key educational pedagogy and curricula. Three types of educational technologies were identified as interventions used to support PBL: learning software and digital learning objects (video/3D models), IWBs and plasma screens, and LMSs. Three types of technologies were selected based on their relatively frequent implementation and innovations as indicated in on-site visitations and communications with health sciences PBL curricula across the globe. Regarding comparisons, although studies adopting experimental designs were included, this was not considered an exclusive criterion given that much educational research in the field is case-based. Finally, included studies indicated outcomes of the effects, both positive and negative, of the use of educational technologies on student learning and staff engagement in PBL. Evidence was determined from both databases and the grey literature.

### Selection of Publications

**Figure 1:**
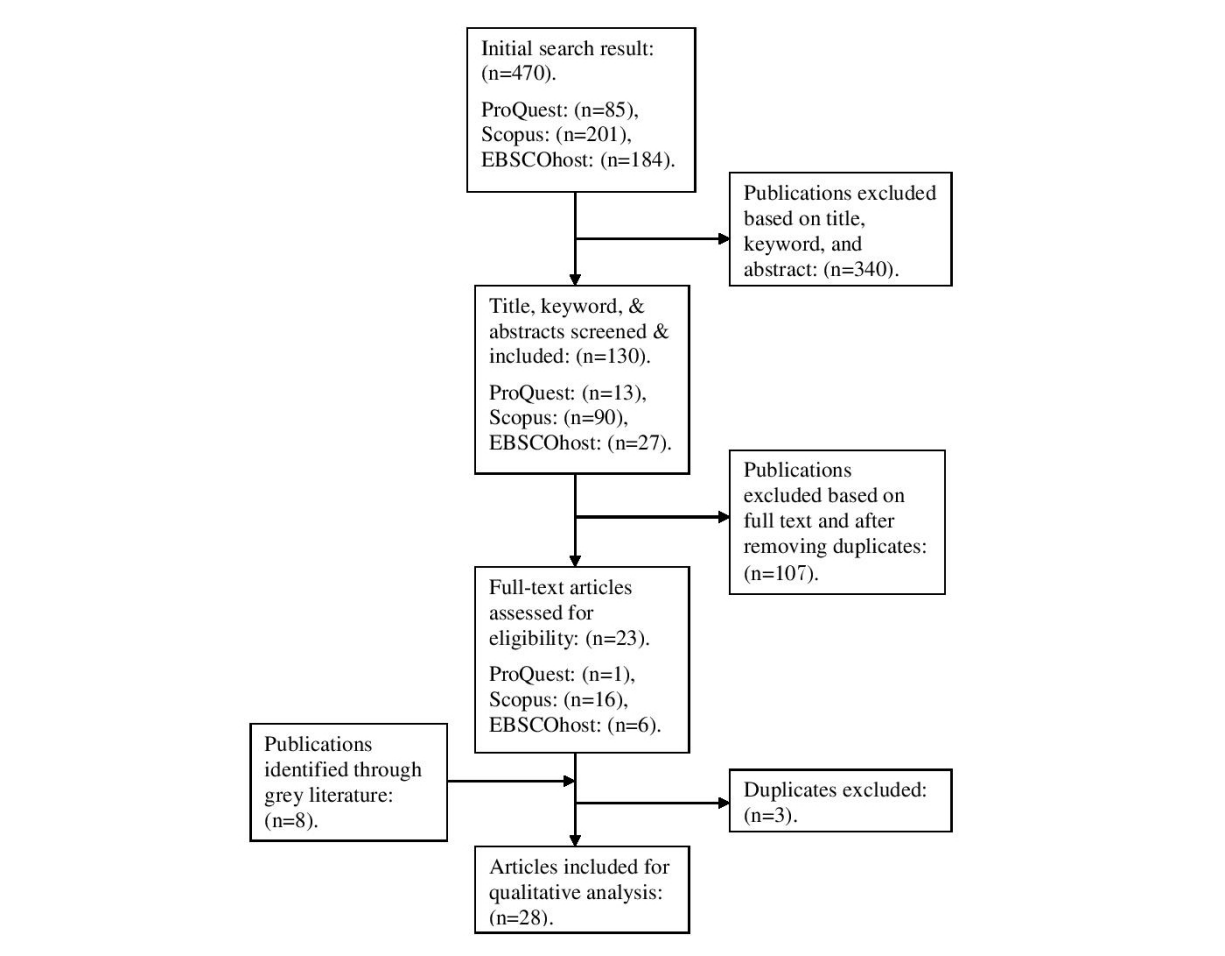
Flowchart of the search process.

**Figure 2:**
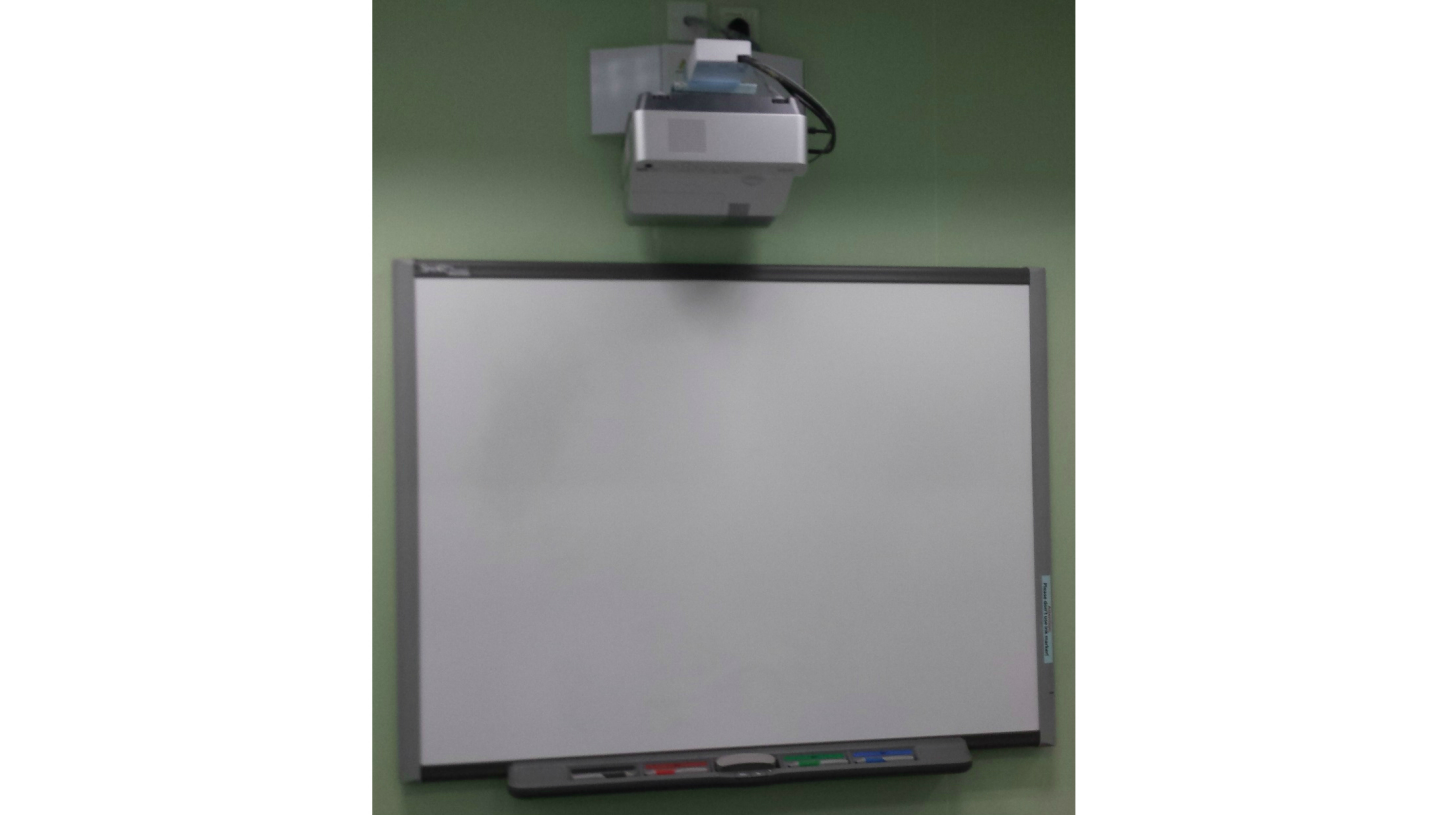
Photo of an interactive whiteboard.

A comprehensive computerized database search of full-text articles published in English from 1996 to 2014 was carried out using 3 education databases: ProQuest, Scopus, and EBSCOhost. Initial search terms were (“educational technologies” OR “learning technologies”) AND (“problem-based learning” OR “problem based learning” OR “PBL”) AND (“clinical” OR “dent*” OR “med*” OR “speech and hearing”). To narrow down the number of studies retrieved in each database, search terms in title/keywords/abstracts were selected in the initial search stage. The titles and abstracts of retrieved papers were first screened and rated for inclusion based on the PICO inclusion criteria. Additional cross-referencing uncovered grey literature in the form of articles and book chapters. Reviews and commentaries were excluded. The review flowchart (Figure 1) indicates the educational database search method and criteria as well as the final number of studies yielded for analysis (N=28). Search results indicate 3 types of educational technologies, learning software and digital learning objects, IWB (Figure 2 and plasma screens, and LMS, were investigated. Given that LMS combines a range of course or subject management and pedagogical tools to offer a means of designing, building, and delivering online learning environments [25], LMS in the search process includes examples of what are also termed course management systems or CMS (eg, WebCT/Blackboard, Angel, Sakai, and Moodle). Following Cook and West’s approach [20], key information (ie, author, year, research design, research purpose, findings) for each article were included. The results were then analyzed and synthesized by narrative or quantitative pooling, exploring effects of educational technologies in PBL-based applications.

## Results

### Overview

Educational technologies have been increasingly used in health sciences education to support or substitute traditional didactic approaches to teaching and learning with inquiry-based approaches. Of the final 28 studies, 20 examined applications of various software and digital learning objects, 5 studies examined the application of IWBs and plasma screens, and 3 explored the application of LMSs in PBL across the 3 clinical disciplines.

### Learning Software and Digital Learning Objects

Table 1 indicates studies that have implemented, evaluated, or explored a variety of software and digital learning objects in problem-based clinical education. Software included an interactive distance learning program in obstetrics and gynecology [26], concept mapping [27], 3D visualization [28], CD-ROM (ErgoROM [29]), a text and image database on CD-ROM [30], a Web-based learning portfolio (SkillsBase [31]), online virtual/simulated patients [32-36], video case(s) [37,38], online cases [39,40], an online resource simulating surgical clinical decision making (SURGENT [41]), Interactive Case-based Online Network [42], virtual PBL [43], and computer-based software [44,45]. The majority of these studies analyzed questionnaire data to investigate user perceptions of the efficacy of the various software and digital learning objects that were piloted, implemented, or developed as learning innovations. The purpose of integrating these software and digital learning objects in PBL were reported variously as an aid/supplement [39] or a replacement [34] for traditional formats, such as lectures, dissection, and clinical practice; or for the development of innovative approaches, bridging the gap between theoretical knowledge and clinical practice [29]; or for facilitating collaboration outside of the classroom [42]. Additional implementation goals were the reinforcement of knowledge construction and supporting decision-making processes [33,41], as well as the advancement of teaching and learning [35].

Perceived positive educational impact was seen in providing a more authentic learner environment [33]; conveying and facilitating understanding of information and complex phenomena [28,41]; facilitating enhanced knowledge [35]; improving cognitive, metacognitive, affective, and overall learning processes or outcomes in PBL [37,38]; having a positive impact on active learning [29,34] and critical thinking [29]; a reflective aid to learning clinical skills [31]; providing a suitable environment for collaboration and communication [33,43]; permitting reduced laboratory time; and increasing small-group activity with less reliance on staff [43]. Although the majority were positively disposed toward learning technologies in PBL, 1 study was more critical [32] finding the PBL video scenario to be cumbersome and not imitating real life; therefore, it was seen to be of little educational value.

The key implications include the importance of the modality of the scenario presentation [32] and the need for guiding principles and a direct facilitator connected to the use of 3D visualizations [28]. Hege et al [39] indicated integration of a computer-based learning tool into the curriculum is as important as the optimization of the software itself and concluded that a few aspects or strategies needed to be considered in integration of software into curriculum (eg, the software should be easy-to-use, highly accessible, and should support user evaluation, the delivering of content, user support, and case maintenance). Jha and Duffy [26] proposed 10 “golden rules” from an evaluation of a CD-ROM program in continuing medical education.

**Table 1:** Studies examining the application of learning software and digital learning objects.

Study	Research design	Research purpose	Findings
Antoniou et al (2014) [36]	The user experienced a specific periodontology case and was asked to provide the optimal responses for each of the challenges of the case. A focus group of 9 undergraduate dentistry students experienced both the Web-based and the Second Life version of this virtual patient.	To assess the suitability of the Second Life MUVE as a virtual patient deployment platform for undergraduate dental education and to explore the requirements and specifications needed to meaningfully repurpose Web-based virtual patients in MUVEs.	The best practices of experiential and immersive game design should be organically incorporated in the repurposing workflow.
Chi et al (2014) [37]	A retrospective cohort study of University of Washington School of Dentistry predoctoral dental students (N=247). The experimental group consisted of first-year dental students (n=63) who received a video case. The historical control group consisted of second-, third-, and fourth-year dental students (n=184) who received a paper case during their first year. A 19-item online survey was administered to all enrolled predoctoral dental students in May 2011.	To compare outcomes associated with video and paper cases used in an introductory public health dentistry course.	Compared to paper cases, video cases significantly improved cognitive, affective, and overall learning outcomes for dental students.
Schwarz et al (2013) [35]	The latest development in the Medical Faculties Network was designed for indexing metadata about simulation-based learning objects. A questionnaire was used to explore students’ attitudes and interests in using the interactive algorithms as part of their health sciences studies.	To present and evaluate simulation-based tools for teaching and learning acute medicine issues.	The peer-reviewed algorithms were used for conducting PBL sessions in general medicine and in nursing. The feedback from the survey suggests that the interactive algorithms are effective learning tools to facilitate enhanced knowledge in the field of acute medicine.
Rampling et al (2012) [32]	A total of 24 students tried the scenario and gave feedback via an online survey.	To create a simulated patient with psychosis for psychiatric training within the online virtual environment of Second Life. After design and delivery of the scenario, medical students were asked to complete it and provide feedback.	The scenario was cumbersome, did not imitate real life, and was of little educational value. Multimedia representations of psychotic symptoms were more positively received and there may be scope for further development.
Bridges (2009) [27]	An intervention was conducted with Year 1 students through workshops that introduced the principles of concept mapping software. Qualitative ethnographic data included Year 1 group concept maps and content-specialist feedback on these. Quantitative data were collected using an online questionnaire.	To reports an intervention to improve both the quality of the concept mapping and submission process.	Students have improved in identifying concepts and relationships and that their maps have increased in complexity and are also more clearly presented. After workshops and trialing, concept mapping has positively affected their thinking processes and improved knowledge retention.
Conradi et al (2009) [33]	A virtual patient (VP) scenarios were designed for learners on a Paramedic Foundation Degree within the virtual world Second Life. A player using the MedBiquitous VP international standard allowed cases to be played both within Second Life and on the Web.	To describe and test the PREVIEW project, trailed a replacement to traditional paper PBL with VPs delivered through a virtual world platform.	The Second Life environment engages students effectively in learning, despite some technology barriers. Students perceived SL could provide a more authentic learner environment than classroom-based PBL.
Poulton et al (2009) [34]	A PBL module was converted to VPs, and delivered to 72 students in 10 tutorial groups, with 5 groups each week receiving VPs with options and consequences, and 5 groups receiving online VPs but without options.	To describe and evaluate the replacement of paper cases by interactive online VPs in PBL.	The replacement of paper cases by online interactive VPs was perceived as a success by students and tutors, and both groups appreciated the improvements in PBL process.
Corrigan et al (2008) [41]	Evaluation was in both a qualitative and a quantitative fashion. A postcourse survey of medical students was used to evaluate access, process, and outcome criteria. SURGENT was used by 98% of students, with 69% spending more than 30 minutes per session on the program.	To assess the introduction of a Web-based module to complement traditional surgical undergraduate curricula.	A Web-enhanced interactive surgical module in an undergraduate course can successfully convey information and understanding beyond the textbook. SURGENT will supplement textbooks and ward experience, allowing students to develop their clinical decision-making skills.
Silen et al (2008) [28]	Questionnaires were used to investigate the medical and physiotherapy students’ opinions about the different formats of visualizations and their learning experiences.	To support learning efficacy by developing and using 3D datasets in health sciences curricula and enhancing the knowledge about possible educational value of 3D visualizations in learning anatomy and physiology.	It was successful to implement 3D images in existing themes in programs. The results show that deeper knowledge is required about students’ interpretation of images/films regarding learning outcomes. There is also a need for preparations and facilitation principles connected to the use of 3D visualizations.
Hege et al (2007) [39]	Different integration strategies of e-learning are presented and compared regarding motivational aspects and acceptance of students and instructors.	To describe and compare different implementation strategies of case-based learning as an important component of e-learning.	A voluntary integration strategy combined with exam relevance of the content.is recommended. The assets and drawbacks of all described strategies are discussed.
Balslev et al (2005) [38]	11 residents were each assigned to 1 of 2 groups. Both groups analyzed an identical vignette of a patient case. Immediately after, 1 group watched a 2.5-minute video recording and the other group read a description of the same video recording. The groups then reanalyzed the case. Thinking processes were tapped by recording and analyzing the verbal group interaction.	To investigate whether adding a brief video case instead of an equivalent written text improves the cognitive and metacognitive processes of residents in PBL.	The verbal interaction showed statistically significant improvements in data exploration, theory building, and theory evaluation after the video case.
Nathoo et al (2005) [42]	Describe a case study of distinct, small-group tutorials over 2 years as part of the Human Nervous System and Behavior course at the Harvard Medical School. Students and faculty were interviewed following completion of the course and their utilization of the system was recorded and examined.	To assess the introduction of a Web-based innovation in medical education that complements traditional PBL curricula. Utilizing the case method as its fundamental educational approach, the Interactive Case-based Online Network allows students to interact with each other, faculty, and a virtual patient in difficult neurological cases.	This is the first study of the Interactive Case-based Online Network learning system in undergraduate medical education, a platform designed to facilitate collaboration outside of the classroom. Data on user perceptions and system utilization suggest that both faculty and students chose to adopt this online learning system as a means for collaboration.
Hudson (2004) [45]	Third-year medical undergraduates at Adelaide University, South Australia were randomly assigned to 4 groups. Following a pretest, only students in the didactic, problem-based, and free-text groups had 2 weeks of free access to a neuroradiology CAL. Learning was quantified by comparing the post- to pretest scores for each of the 4 groups.	To test the hypothesis that a Computer-aided learning (CAL) tutorial, will result in superior learning (ability to apply and retain knowledge) to that obtained in more passive CAL formats.	While users of an interactive CAL tutorial demonstrated significant learning outcomes compared to non-CAL users, these outcomes were not superior to those achieved from noninteractive CAL.
Sibbald (2004) [40]	Students performed 3 PBL exercises for the same topic. Educational outcomes and students perceptions from an online survey are reported.	To encourage self-directed skill development; addresses learning style preferences; quantitatively and qualitatively assesses the relative advantages of electronic-based vs traditional PBL tools on knowledge and skills building.	This project gave students a balanced, enhanced knowledge perspective from 3 PBL formats; promoted peer teaching, mentoring, and technology skills; and provided insights comparing Web-based tools to other methods for autonomous lifelong learning.
August-Dalfen & Snider (2003) [29]	The program ErgoROM was used with a group of third-year occupational therapy students (n=50) in a semester-long course at McGill University in Canada.	To explore the students’ perceptions of using the ErgoROM and impact of the CD-ROM learning experience on their active learning and critical thinking skills.	Overall, 91% of respondents rated the ErgoROM as either “excellent” or “very good.” Additionally they reported that ErgoROM had a positive impact on active learning and critical thinking.
Bowdish et al (2003) [43]	A quasi experimental, posttest-only research design compared the virtual problem-based learning (VPBL) and a text-based version of the same PBL exercise on students’ achievement, as measured by a set of selected physiology examination items, and their perceptions of the learning environment, as measured by the Teaching and Learning Environment Questionnaire.	To report the results and insights of an exploratory investigation of the effectiveness of a prototypic virtual problem-based learning (VPBL) exercise delivered via the Web that uses Hypermedia Assisted Instructional Technologies.	The VPBL is equally as effective as the text-based version for enhancing students’ learning and their learning environment in small-group PBL sessions.
Dornan et al (2003) [31]	Qualitative analysis of users’ requirements and development of a Web-based learning portfolio. Direct observation of users during a “think-aloud” protocol, a validated software users’ measurement inventory, and a questionnaire designed to test whether SkillsBase met its users’ requirements.	To evaluate the use of information and communications technology to present a curriculum of clinical skills in a user-friendly format.	SkillsBase meets the design specification for a training and reflective aid to learning clinical skills and is very usable.
Jha & Duffy (2002) [26]	The questionnaire was sent out to 150 Distance Interactive Learning in Obstetrics and Gynaecology (DIALOG). The qualitative data obtained from the evaluation resulted in 10 common items being identified by the majority of respondents.	To carry out a formative evaluation of DIALOG to determine whether DIALOG was achieving its educational objectives.	One of the main benefits of formative assessment is to determine what needs to be done to maintain or improve the program in future. 10 “golden rules” emerged from the evaluation of DIALOG.
Levine et al (1999) [44]	Success of utilization was measured by quantitative improvements in student perceptions and attitudes over a 3-year period.	To describe and assess the implement and integration of computer-based activities into a problem-based gross anatomy curriculum.	The data suggested increasingly positive and beneficial student attitudes toward educational technology, for networks as a faster and more effective method of student/faculty communication, and in the utilization of computer-based instruction for greater flexibility and efficiency in learning the laboratory material.
Andrew & Benbow (1997) [30]	A brief questionnaire was given to a random sample of 100 students at the beginning of the last lecture of the third-year lecture course.	To describe and evaluate the conversion of a traditional image archive into an image resource on compact disc.	This resource useful as an aid to revision, despite relative computer illiteracy, and it is anticipated that students on a new PBL course that incorporates experience with information technology will benefit even more readily when they use the database as an educational resource.

### Interactive Whiteboards

Table 2 indicates that the use of IWBs is a new phenomenon in clinical education with 4 studies of IWBs in PBL curricula [3,11,46,47] arising in the past 7 years in addition to 1 study on the use of plasma screens [48] in 2005. Kerfoot [48] indicated that the introduction of computers and plasma screens had a positive impact on PBL tutorials. Bridges and her colleagues [11] adopted an interactional ethnographic methodology to analyze student engagement with digital materials through the use of an IWB and found that “the integration of face-to-face and virtual modalities through the single PBL group’s use of an IWB across tutorials and self-study was seamless and supported whole-group engagement in the process.” In another study in undergraduate dentistry, Bridges and her colleagues [3] noted that the use of different texts and tools in one problem cycle supported a discursive shift from stimulus for hypothesizing to evidence for final hypotheses. Lu’s 2 studies in medical education [46,47] compared a traditional whiteboard group with an IWB group . One study [47] described that nature of scaffolding of collaborative problem solving under the 2 conditions and concluded that educational technology such as IWB can help by expanding the scaffolding choices. The other study [46] identified relationships between learners’ collaborative decision making and communicative discourse when engaged in a simulated medical emergency. Group differences were found in that IWB group participants engaged in more adaptive decision-making behavior earlier than the traditional whiteboard group, which led to shared understandings and subsequently to more effective patient management [46]. They also found more productive argumentation in the type of collaborative discourse produced in IWB medical student groups [46]. There are limited studies to show the relative advantages or disadvantages of using IWBs in health sciences education. More studies, therefore, are encouraged to explore both the impact of using IWBs for large-screen visualization and collaboration.

**Table 2:** Studies examining the application of interactive whiteboards and plasma screens.

Study	Research design	Research purpose	Findings
Bridges et al (2012) [3]	Case study of a single third-year PBL group (n=8) as they engaged in learning activities across a problem cycle. The study investigated the data trail across PBL learning events and contexts and the various discourse members. One discourse member of the PBL group was selected as an anchor point for tracking across the data collection.	To investigate PBL-as-process in clinical education through detailed analysis of the “way” students and their tutors construct knowledge and negotiate meaning in situ in a dental PBL curriculum.	The way students experience and understand 2 “black box” facets of their PBL learning, independent learning, and online learning, are explored by adopting interactional ethnography multimodality within a theory of semiotics examines multimodal texts support cognition and transformative learning.
Bridges et al (2010) [11]	IWBs were installed in all PBL tutorial rooms and IT support was provided for all students and facilitators in a 5-year undergraduate dental PBL curriculum. Year 1 students (n=55) received additional workshops and IWB support. Analysis of 4 hours of video-recorded learning activities undertaken by a Year 1 undergraduate PBL group (n=8) on 3 occasions during the same week.	To establish and evaluate how the progression from a digital repository approach toward an interactive blending of technology within face-to-face tutorials might be supported and resourced.	The group accessed a range of digital materials to support learning within and across all phases of a problem cycle. The use of learning objects and online resources within a problem cycle supported inquiry learning and the discursive shift in student talk from a stimulus for hypothesizing in relatively lay terms in the first tutorial to evidence. The integration of face to-face and virtual modalities through the use of an IWB within the tutorial was seamless and supported whole-group engagement in the problem process.
Lu et al (2010) [47]	2 conditions (traditional whiteboard and IWB) using a “deteriorating patient” case were examined. 2 groups of third-year medical students in the Department of Internal Medicine in a large urban teaching hospital volunteered to participate.	To describe the nature of scaffolding of collaborative problem solving under 2 conditions—with technological support and without.	Although appropriate scaffolding is still based on the teacher’s domain knowledge and pedagogy experience, technology can help by expanding the scaffolding choices that an instructor can make in a medical training context.
Lu & Lajoie (2008) [46]	2 groups of 7 third-year medical students volunteered to participate. Both groups were asked to solve a learning activity. 1 group of students used a traditional whiteboard and the other used an IWB while solving the patient problem. 2 kinds of data were collected: collaborative decision-making discourse and computer records of whiteboard annotations.	To investigate the collaborative decision making and communicative discourse of groups of learners engaged in a simulated medical emergency across the 2 condition subgroups.	IWB enabled data sharing and construction of shared understandings about the patient. Shared visualization clarified verbal interaction, promoted productive argumentation, and facilitated negotiation. Argumentation tools embedded into the IWB design enhanced groups’ decision making and communicative interactions in the simulated medical emergency.
Kerfoot et al (2005) [48]	37 tutorial groups, were observed to record the patterns of use of the computers and plasma screens. Based on these observations, surveys were developed and distributed to students and tutors.	To examine how the introduction of this educational technology impacted PBL tutorials.	Both students and tutors reported that the introduction of computers and wall-mounted plasma screens had positively impacted tutorials. Questions were raised as to how this technology might alter tutorial dynamics.

### Learning Management Systems

Table 3 lists studies that adopted various LMSs to support problem-based approaches. These included the application of WebCT [49], VMS Portal [50] to manage inquiry-based materials and activities for PBL curricula, and iSUS for self-directed learning [51].

A few studies noted the positive effects of using LMS in PBL curricula [49-51]. In Dornan’s study [51], a Web-based LMS-ISUS helped to provide practical guidance about what to learn and how to learn, helped access appropriate experiences and manage time, gave feedback on students’ accumulated real patient learning, provided peer comparison, and helped self-management. The authors argued that ISUS can provide the motivational jigsaw to fill the gap between PBL and placement learning. McLean [49] found students perceived that LMS are the most useful means of communication and resource delivery for PBL in medicine. His study also highlighted limitations in that insufficient support, resources, and training might result in less successful implementation of educational technologies.

With regard to the question of scaffolding, some of the previous studies noted that monitoring, support, and development are important for efficient and positive implementation of an LMS in PBL curricula. In the VMS Portal project [50], medical students were involved in website development to help, consolidate, integrate, and develop Web resources for their peers. Critical to successful scaffolding in PBL are tutor facilitation strategies [10]. In online environments, tutor presence, ongoing engagement, and timely feedback become factors in facilitating students’ problem solving, self-directed learning, and collaboration in health sciences education.

**Table 3:** Studies examining the application of learning management systems.

Study	Research design	Research purpose	Findings
Rosenbaum et al (2009) [50]	VMS Portal used software and database technology for a highly customized Web portal for medical students. Access to course material, evaluations, academic information, and community assets were customized for individual users. Modular features were added in response to student requests and feedback and monitoring of usage habits.	To describe a practice-based focus by 2 medical students to create a website for all medical students.	Medical students are uniquely positioned to help consolidate, integrate, and develop Web resources for peers. As other medical schools create and expand digital resources, input by medical students should be solicited.
Dornan et al (2005) [51]	A Web-based LMS included 66 placement students in a PBL medical curriculum. Data were free-response comments from 16 students during 7 weeks of usage, transcripts of pre- and postgroup discussions, and questionnaire responses (100%).	To establish whether and under what conditions medical students can learn in a self-directed manner in the clinical environment.	Students valued affective and pedagogic support, and relied on teachers to manage their learning environment. With support, they were motivated and able to choose how and when to meet their learning needs.
McLean & Murrell (2002) [49]	WebCT in PBL, student-centered curriculum was introduced. A survey was conducted after completing the first module in School of Medicine in South Africa.	To gather user feedback with regard to the value of WebCT as a curriculum support, especially the value of WebCT for the delivery of digitized material.	Students responded positively to the communication facility. WebCT will be particularly useful when students are off campus, undertaking electives and community service.

## Discussion

### Principal Findings

The journal articles and book chapters examined in this systematic review indicate the generally positive effect of the thoughtful implementation of educational technologies in PBL. This is particularly the case where such technologies support scaffolding thereby reducing cognitive load and allowing students to learn in complex domains [19]. Firstly, when considering resource development, educational technologies enable provision of rich, authentic problems and/or case contexts accessible on demand in virtual spaces. Online virtual/simulated patients, video case(s), and online cases [33-41] convey complex phenomena in a more authentic learning environment. Secondly, educational technologies provide not only access to engagement in the problem-based inquiry, but also structure information by embedding expert knowledge and skills. This may be in the form of problem-relevant videos and simulations made available during self-directed learning [3] demonstrating case reports independent of time and place. Thirdly, educational technologies support students and their facilitators in making disciplinary thinking explicit. Dedicated software can help learners to construct explanations, structure tasks, and make them more manageable [19]. Adaptations of standard LMS as well as dedicated inquiry-based LMS can provide a platform to elicit articulation, collaboration, and reflection [52]. Technological resources, such as different software and 3D models, LMS, and IWB, can assist students engage in problem-solving processes.

Although generally positive, a limited number of studies have indicated the adverse effects of educational technologies or their methods of implementation. In this review paper, the less successful implementations may be attributable to the content or delivery of the video scenario [32]. Additional cognitive burden due to higher levels of complexity was seen as a possible limitation to their effective implementation; however, in highly positive cases, technologies were seen as providing an additional supportive scaffold for student learning. In terms of infrastructure and staff development, insufficient support, resources, and training [49,53] were seen as disabling. Adopting and/or adapting functionalities from software or LMS with limited staff-student and/or student-student interactivity and limited student feedback processes were seen as shortcomings in cases where this occurred. This would indicate that thoughtful instructional design approaches need to be applied when adapting more traditional systems or when designing new programs for inquiry-based learning [54]. Well-designed empirical research studies are needed to establish best practices for technological hardware and software in enhancing teaching and learning productivity and building stronger learning communities [55].

Although the major forms of educational technologies presented in these studies look very promising and are potentially fit for educational purpose in many problem-based health sciences education settings, there is a need for support and training in light of the ever-changing nature of both technical knowledge of teaching staff and the technological affordances themselves. The power of computers and Internet, merging with multimedia and interactive spaces, appear to allow a high degree of flexibility and accessibility to digital instructional content in health sciences education [56]. These affordances may enable integration of technologies into the curriculum and support staff development by encouraging interactive teaching and learning. However, the often-false assumption is the “sink or swim” approach in which faculty may assume students’ prior technological skills or knowledge. Where new affordances are introduced or even old affordances with new inquiry-based purposes, additional hands-on tutorials and training are helpful to facilitate student adaptation to the technology and ensure optimal benefit [49,57]. Successful multimedia teaching and learning relies not only on the proper use of information technology, but also on a clear implementation strategy [58]. Implications also arise in considering Technological Pedagogical Content Knowledge [59] with regard to disciplinary influences on the ways that educational technologies are incorporated into the curriculum. Guidance and support should be tailored to meet the needs of each user including learner, facilitators, and curriculum developers to be at its most effective.

### Conclusions

In conclusion, this literature review indicates a generally positive effect from the adoption of various educational technologies in PBL. Positive outcomes for student learning included providing rich, authentic problems and/or case contexts for learning; supporting student development of medical expertise through the accessing and structuring of expert knowledge and skills; making disciplinary thinking and strategies explicit; providing a platform to elicit articulation, collaboration, and reflection; and reducing perceived cognitive load. Insufficient technical support, infrastructure, and resources were seen as impacting negatively on uptake and learning outcomes. Staff and student induction and ongoing training in the use of educational technologies for learning in inquiry-based contexts such as PBL is recommended.

Although educational technologies have been increasingly used in health sciences education, it has been questioned whether they can completely substitute traditional teaching methods [60]. The rise of Massive Online Open Courses in all fields, including health sciences, has been seen as positive, particularly for continuous medical education and public health literacy [15]. In considering undergraduate inquiry-based curricula, this review supports Hmelo-Silver [19] and Bridges et al’s [61] predictions that technology can play an important but synergistic role with other components of PBL. Further research into the various applications of educational technologies in PBL curricula is needed to fully realize their potential in enhancing inquiry-based approaches in health sciences education. In an increasingly digital, networked world, convergence of educational technologies is increasingly apparent. This has given rise to understandings that learners are positioned within digital ecosystems. Consequently, it is possible that a learner might engage with the merging of distinct educational technologies. The effects of learning in a digital ecosystem need to be identified and explored in further research.

## Abbreviations

3D: 3-dimensional
CAL: computer-aided learning
CMS: course management systems
DIALOG: Distance Interactive Learning in Obstetrics and Gynaecology
IWB: interactive whiteboard
LMS: learning management system
PBL: problem-based learning
PICO: population, intervention, comparison, and outcomes
VP: virtual patient
VPBL: virtual problem-based learning
